# Bayesian estimation of genomic copy number with single nucleotide polymorphism genotyping arrays

**DOI:** 10.1186/1756-0500-3-350

**Published:** 2010-12-30

**Authors:** Beibei Guo, Alejandro Villagran, Marina Vannucci, Jian Wang, Caleb Davis, Tsz-Kwong Man, Ching Lau, Rudy Guerra

**Affiliations:** 1 Department of Statistics, Rice University, 6100 Main, Houston, TX 77005-1827, USA; 2 Department of Statistics, University of Connecticut, Storrs, CT 06269-4120, USA; 3 Department of Pediatrics, Division of Hematology-Oncology, Baylor College of Medicine, Texas Children's Hospital, 6621 Fannin St., MC 3-3320 Houston, TX 77030, USA; 4 Structural and Computational Biology and Molecular Biophysics Program, Baylor College of Medicine, One Baylor Plaza, Houston, TX 77030, USA

## Abstract

**Background:**

The identification of copy number aberration in the human genome is an important area in cancer research. We develop a model for determining genomic copy numbers using high-density single nucleotide polymorphism genotyping microarrays. The method is based on a Bayesian spatial normal mixture model with an unknown number of components corresponding to true copy numbers. A reversible jump Markov chain Monte Carlo algorithm is used to implement the model and perform posterior inference.

**Results:**

The performance of the algorithm is examined on both simulated and real cancer data, and it is compared with the popular CNAG algorithm for copy number detection.

**Conclusions:**

We demonstrate that our Bayesian mixture model performs at least as well as the hidden Markov model based CNAG algorithm and in certain cases does better. One of the added advantages of our method is the flexibility of modeling normal cell contamination in tumor samples.

## Background

Gene dosage variations occur in many diseases, as well as in normal populations (e.g., [1,2]). In cancer, copy number losses and gains are known to contribute to alterations in the expression of tumour-suppressor genes and oncogenes, respectively, see for example [3,4]. Developmental abnormalities, such as Down, Prader Willi, Angelman and Cri du Chat syndromes, result from gain or loss of one copy of a chromosome or chromosomal region. Thus, detection and mapping of copy number abnormalities provide an approach for associating aberrations with disease phenotype and for identifying critical disease-causing genes. As an example, Rendon et al. [5] constructed a first-generation copy number variation (CNV) map of the human genome through the study of 270 HAPMAP individuals from four populations with ancestry in Europe, Africa or Asia, [6]. A total of 1, 447 copy number variable regions (CNVRs), covering 360 megabases (i.e., 12% of the genome), were identified in this study. These CNVRs contained genes, disease loci, functional elements and segmental duplications.

DNA from the individuals in the study of [5] was analyzed for CNV using two technologies: single-nucleotide polymorphism (SNP) genotyping arrays, and comparative genomic hybridization (CGH). Array-based Comparative Genomic Hybridization (aCGH) is a molecular-cytogenetic method for the analysis of DNA copy number changes [1]. The method is based on hybridization of fluorescently labeled tumor DNA and reference DNA on a microarray platform containing Bacterial Artificial Chromosome (BAC) clones or spotted DNA. As a gold standard, it is robust in identifying long segments of chromosomal alterations. However, although the resolution of aCGH has been improved, it is still not high enough to detect amplifications or deletions of relatively short segments, [7] and [5]. The high-density SNP array, which can accommodate hundreds of thousands of SNP probe sets simultaneously, is an alternative approach to detect genome wide copy number aberrations which has much higher resolution than CGH, see [8]. Compared to CGH, SNP array based experiments are newer and are becoming more popular for copy number analysis.

A number of statistical methods have been proposed to estimate copy numbers from various platforms. Two of the most popular methods for SNP arrays are dchip and Copy Number Anlyser for GeneChip (CNAG). Zhao et al. [9] proposed dChip, an algorithm that derives model-based estimates of SNP copy numbers that incorporate probe effects and a hidden Markov model (HMM) to infer integer-valued copy numbers. Although the current version of the dChip software can accommodate the newer SNP arrays, such as the Affymetrix 250K array, it is not optimized for it. Nannya et al. [10] developed the CNAG algorithm, which accounts for the length and GC content of the PCR products. Accounting for the length and content of GC elements seems to improve copy number inference [10]. Another source of variation that can affect a copy number analysis is the so-called "genome wave" [11,12], a genome-wide spatial autocorrelation pattern in signal intensity. Since the genome wave may be confounded with the copy number profile across a chromosome, investigators should examine their intensity data for its presence and adjust the data accordingly. Since the genomic wave [11] is thought to be in large part due to GC content, the CNAG algorithm can also be thought of as an adjustment for wave effects possibly present in SNP array data. Again, an HMM is used to infer integer copy numbers. The HMM approach can also be found in the algorithms underlying QuantiSNP [13] and PennCNV [14], both of which use the log-R-ratio and *B*-allele frequency to infer the copy number state of each SNP. These two methods consider a six-state Markov model which distinguishes copy-neutral loss of heterozygosity from the normal state. Most HMM based algorithms use the Viterbi algorithm [15] to infer integer copy numbers.

To date, there are a handful of Bayesian methods for copy number inference. Most are for CGH data, but a few exist for SNP data. Rueda and Diaz-Uriarte [16] proposed RJaCGH, a nonhomogeneous HMM in a Bayesian context for CGH data. Instead of prespecifying the number of states as a conventional HMM, a reversible jump Markov Chain Monte Carlo (MCMC) method is used to allow for varying numbers of hidden states. Bayesian model averaging is used to obtain final estimates. Pique-Regi et al. [17] developed a method called Genome Alteration Detection Algorithm (GADA) that is based on sparse Bayesian learning [18]. The approach takes advantage of the a priori assumption that the number of copy number alterations (break points) is sparse with respect to the number of probes. As with several other methods, advantage is also taken of the fact that the copy number pattern across a chromosome can be modeled as a piecewise constant function or vector. The GADA output gives copy number results in the form of a segmentation, viz., a collection of ordered segments defined by their breakpoints and amplitudes. To obtain integer-valued copy numbers or alteration status (loss, normal, gain), the estimated segments must be analyzed by a thresholding procedure, such as Huang et al. [19]. GADA can be applied to both CGH and SNP based data. Rancoita et al. [20] also make use of piecewise constant modeling in their algorithm, mBPCR, which is a modification of the original Bayesian Piecewise Constant Regression (BPCR) method developed by Hutter [21]. This method is general for data that take the form of a piecewise constant function with unknown segment numbers, boundaries, and levels. Rancoita et al. illustrate the mBPCR method using SNP data, but it appears that log-ratios based on CGH data can also be analyzed.

In addition to those described above, several other statistical methods have been developed for copy number analysis. They vary in their assumptions, inference (segmentation, alteration status, integer copy number), platform (CGH, SNP), input data (e.g., CEL files or generic normalized log-ratio), and software implementation (e.g., commercial, web-based, customized academic program). Winchester et al. [22] describe and compare a number of methods. No method stands out as uniformly best and Winchester et al. suggest analyzing copy number data with at least two different methods to assess consistency and robustness of results. In this paper, we could not consider many of those methods for performance comparison because most of these algorithms do not estimate integer copy numbers.

Most of the copy number methods assume normalized log-ratios as input. Relatively few include adjustments for known factors affecting inference. GC content and fragment length have been mentioned as factors affecting copy number inference. Another factor from tumor samples is normal cell contamination. Indeed, most tumor samples are heterogeneous and include both cancer cells (with copy number aberrations) and normal cells. The larger percentage of normal cells present, the more difficult it is to infer copy number aberrations in the tumor cells; the log-ratios tend to shrink to the null value of zero. None of the above methods implement an adjustment for normal cell contamination. Below we show how our proposed method can account for this factor.

Here we propose a Bayesian spatial normal mixture model for inferring SNP-based integer copy number. Bayesian mixture models were used by [23] for CGH-based copy number estimation. There the authors considered a three-state (loss/normal/gain) mixture and introduced a spatial structure to reflect correlated segments (e.g. BACs). Spatial correlation was induced through the weights of the mixture via Markov random fields. In our approach, instead of considering three states, we allow for an unknown number of mixture components and achieve inference using a reversible jump Markov chain Monte Carlo method. As in [23] we use Markov random fields to account for correlated neighboring SNPs. In contrast to models that incorporate HMMs to infer integer copy numbers, our modeling approach uses information (neighboring SNPs) on both sides of a SNP. In addition, we account for cell contamination by shrinking the theoretical copy number log-ratios towards zero. The implementation only requires ordered (normalized) log-ratios and, therefore, may be applied to data from any platform suitable for copy number estimation. In Section 2 we present the model and method of inference. Section 3 reports on a simulation study and application to real data. The real data study includes cases where cytogenetics has shown large regions of gain or loss and we also show novel smaller regions detected by our algorithm. The new aberrations are validated by CGH and/or PCR. A discussion is given in Section 4 and an Appendix provides details on the MCMC algorithm.

## Methods

### Model

Let *y_i _*be the preprocessed log-ratio of SNP *i *ordered along the chromosome. Following the notation of [24], we consider a normal mixture model with *k *unknown components corresponding to *k *copy numbers,

(1) $$p(y_{i}|k,\omega ,\mu ,\sigma^{2})=\sum_{j=1}^{k}\omega_{ij}N(y_{i}|\mu_{j},\sigma_{j}^{2})$$

where ***μ ***= (*μ*_1_,..., *μ_k_*) and $\sigma^{2}=(\sigma_{1}^{2},\ldots ,\sigma_{k}^{2})$ represent the vectors of means and variances of the *k *components. The matrix of weights ***ω ***= (*ω_ij_*) is such that 0 ≤ *ω_ij _*≤ 1 and $\sum_{j=1}^{k}\omega_{ij}=1$ for all *i*. In our application the components represent the true copy numbers (i.e. copy number equals to 0, 1, 2, 3,...). Given a chromosome with *n *SNPs, let *z*_1_,...*z_n _*be the allocation variables, indicating to which mixture component SNP *i *belongs to. These are marginally distributed according to a multinomial distribution with

(2) $$p(z_{i}=j|k,\omega ,\mu ,\sigma^{2})=\omega_{ij},$$

for *j *= 1,...,*k*. Since copy number aberrations tend to occur over contiguous segments, we impose that neighboring SNPs have similar multinomial probabilities of belonging to the copy number classes. To this end, for *k *components we introduce *k *independent Gaussian Markov random fields (GMRF), **x***_j _*= (*x_ij_*, *i *= 1,...,*n*), see [24] and [23], each with joint distribution

(3) p(xj|h)=c(h)×exp{−12(h∑i~i′(xij−xi′j)2+∑i=1nxij2)}

where Σ_*i~i' *_denotes the sum over all pairs of neighbors and where $c\left(h\right)={\left(2\pi \right)}^{-\frac{n}{2}}{\prod_{i=1}^{n}\left(1+hg_{i}\right)}^{\frac{1}{2}}$, with *g*_1_,...,*g_n _*the eigenvalues of a matrix of coding adjacencies. Since the conditional distribution of *x_ij _*only depends on its neighbors, neighboring *x_ij_*'s will tend to have similar values. The parameter *h *is non-negative and controls this effect: large values of *h *induce smoother realizations in the GMRF, and as *h *→ 0 independent realizations take place. For the weights, *ω_ij_*'s, we borrow spatial correlation from the GMRF's by defining logistic transformations of the type

(4) $$\omega_{ij}=\frac{exp(x_{ij}/\phi )}{\sum_{l=1}^{k}exp\left(x_{il}/\phi \right)},\;j=1,\ldots ,k,$$

where *ϕ *is a scaling factor specified by the user. In the simulation study of Section 3 we investigate robustness of the results to different values *ϕ *of and varying number of neighbors.

### Prior distributions

In this section we discuss the prior distributions for the model parameters, including the number *k *of mixture components, the normal mixture means and variances, and the smoothing parameter *h*.

#### 1. Number of mixture components, k

We choose a truncated Poisson distribution with mean 2 as the prior distribution of the number of components, *k*,

(5) $$k\sim TPoisson\left\{1,\ldots ,k_{max}\right\},$$

with *k_max _*a pre-specified large integer. We take *k_max _*= 7 for illustration purposes, corresponding to copy numbers 0, 1, 2, 3, 4, 5, *and *> 5. Here 7 is arbitrary, and we can use any positive value that makes sense for the data under consideration.

#### 2. Normal mixture means

We deviate from the approach of [24] by constructing *k_max _*uniform distributions, {*ν_j _*= *U *(*a_j_*, *b_j_*), *j *= 1,...,*k_max_*}, and assuming that each component mean *μ_j _*follows one of these uniform distributions independently. The uniform interval boundaries are very important. We choose the intervals to be non-overlapping and to contain the theoretical copy number values. According to [10], the observed mean values for the 7 components without contamination are approximately -1.24, -.49, 0, .365, .657, .899 and 1.106 for copy numbers 0, 1, 2, 3, 4, 5 and > 5, respectively. In this paper results were obtained using the following intervals: (-2, -.8), (-.6, -.25), (-.05, -.05), (.15, .4), (.45, .66), (.75, .9), (.95, 1.3), corresponding to copy numbers 0, 1, 2, 3, 4, 5, > 5, respectively. These intervals are the default values we used in the application and have worked well in most cases. Our results did not show sensitivity to the actual values we used for the extremes of the intervals, i.e., other disjoint sets of intervals worked well too.

*Remark 1*: Due to normal cell contamination, the true log-ratios tend to shrink toward zero, and in practice some degree of normal cell contamination tends to be present. We thus decided to center the uniform distributions closer toward the null value of zero rather than at the theoretical means given above, except for CN = 0 and CN > 5. These exceptions are largely due to where we wanted to locate the respective uniform support; see Remark 3 below.

*Remark 2*: Moving the uniform intervals closer to zero resulted in some of the theoretical means being located close to a uniform boundary. For example, for CN = 5, the theoretical mean of .899 is just inside the right boundary of .9. This does not cause a problem of misclassification since normal cell contamination brings the mean closer to the left boundary.

*Remark 3*: We also varied the length of the uniform intervals since the log scale makes the consecutive theoretical values become increasingly closer to each other; the consecutive pairwise distances between the theoretical means from -1.24 to 1.106 are .75, .49, .365, .292, .242, .207. If the uniform intervals were forced to be of equal length we would have either relatively short non-overlapping intervals or over-lapping long intervals. Since the uniform intervals are not of equal length, the gaps between the intervals are unequal, as well.

In cases where the exact percentage (*p*) of normal cells is known then such intervals can be chosen to contain

$$log_{2}\left[\frac{2p+j(1-p)+b}{2+b}\right],$$

for any copy number *j*, with background factor *b*, see [10], and then choosing the length of the intervals so that the *k_max _*intervals are non-overlapping.

#### 3. Normal mixture variances

We assign an inverse gamma prior distribution to $\sigma_{j}^{2}$. In the application we center this distribution on 0.2 and induce a vague specification by letting the variance be large.

#### 4. Smoothing parameter

We assign *h *a uniform distribution with a wide range, *h *~ *U *(0, *h_max_*), with *h_max _*= 1000, 000, to induce smooth realizations.

We provide further discussion of these prior selections below in Section 3 in the context of the simulations and real data applications.

### Posterior inference

**Figure 1:**
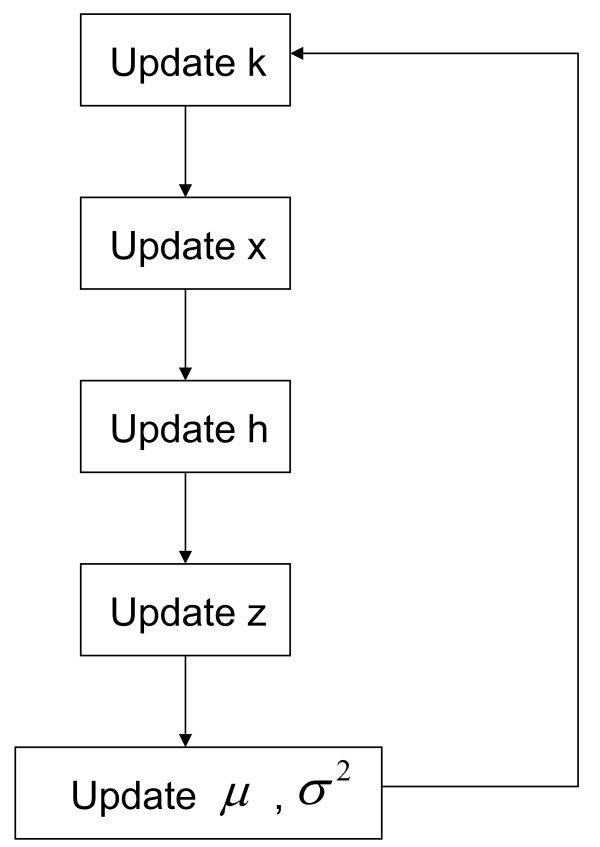
**Flowchart of the MCMC algorithm for posterior inference**. Updating steps on *k*, *μ*and *σ*^2 ^are done at the chromosome level, while those on *x*, *h *and *z *are done at each SNP location. Steps are given in their corresponding order in the implemented algorithm.

We employ MCMC with reversible jump to achieve posterior inference. A flowchart of our MCMC algorithm for posterior inference is given in Figure 1. We note that while updates on *x *and *z *are done at each SNP location *i*, the updating steps on *k*, *h*, *μ *and *σ*^2 ^are global, i.e., done at chromosome level. Steps are described below in their corresponding order in the implemented algorithm. Additional details are given in the Appendix.

**• Updating ***k*: This step causes creation or deletion of components, therefore requiring the sampler to jump between subspaces with different dimensions. To implement the sampler, we use reversible jump MCMC (RJMCMC), see [25] and [26]. We update *k*' = *k *+ 1 with probability *b_k_*, and *k*' = *k *- 1 with probability $1-b_{k}(b_{1}=1,\;b_{k_{max}}=0,\;b_{k}=.5\;\text{for}\;k=2,\ldots ,k_{max}-1)$. If *k*'= *k *+ 1, we draw a new component from the remaining *k_max _- k *components with equal probability, and draw μ_* _from the corresponding uniform distribution. We also draw $\sigma_{*}^{2}$ and x_* _from the prior distributions. We then increase the dimensions of the vector parameters ***μ***'= (***μ***, μ_*_), $\sigma^{2^{\prime }}=(\sigma^{2},\sigma_{*}^{2})$, and **x**'= (**x**, x_*_) and accept the new component with probability:

(6) $$\begin{array}{l} min(1,\frac{\left(1-b_{k+1})\frac{1}{k+1}p(k+1\right)}{b_{k}\frac{1}{k_{max}-k}p(k)}\\ \;\times \prod_{i=1}^{n}\frac{\sum_{j=1}^{k+1}{\omega^{\prime }}_{ij}N(y_{i}\;|\;{\mu^{\prime }}_{j},{\sigma^{\prime }}_{j}^{2})}{\sum_{j=1}^{k}\omega_{ij}N(y_{i}\;|\;\mu_{j},\sigma_{j}^{2})}) \end{array}$$

If *k' *= *k *- 1, we instead randomly pick a component from the discrete uniform distribution on {1,...,*k*} and remove $\mu_{*},\sigma_{*}^{2},x_{*}$ from ***μ***, ***σ***^2^, **x**. Similarly, the acceptance probability is

(7) $$\begin{array}{l} min(1,\frac{b_{k-1}\frac{1}{k_{max}-(k-1)}p(k-1)}{\left(1-b_{k})\frac{1}{k}p(k\right)}\\ \;\times \prod_{i=1}^{n}\frac{\sum_{j=1}^{k-1}{\omega^{\prime }}_{ij}N(y_{i}\;|\;{\mu^{\prime }}_{j},{\sigma^{\prime }}_{j}^{2})}{\sum_{j=1}^{k}\omega_{ij}N(y_{i}\;|\;\mu_{j},\sigma_{j}^{2})}) \end{array}$$

**• Updating x: **We update each location using a Metropolis-Hastings step, see [27] and [28]. We perform these *n *updates sequentially, i.e., we update (*x*_11_,...,*x*_1*k*_) first, then update (*x*_21_,...,*x*_2*k*_), etc.. For each location *i*, we use a proposal distribution of the type

$$\prod_{j=1}^{k}N\left({x^{\prime }}_{ij}|\frac{h\sum_{i^{\prime }∼i}x_{i^{\prime }j}}{1+hn_{i}},\frac{1}{hn_{i}}\right)$$

where *n_i _*is the number of neighbors at location *i*. The acceptance probability is

$$min\left(1,\frac{\sum_{j=1}^{k}{\omega^{\prime }}_{ij}N(y_{i}|\mu_{j},\sigma_{j}^{2})}{\sum_{j=1}^{k}\omega_{ij}N(y_{i}|\mu_{j},\sigma_{j}^{2})}\right)$$

where ***ω***'are the weights associated to the proposed **x**.

**• Updating ***h*: We use a Metropolis-Hastings random walk with a proposal defined by a truncated normal distribution, $h^{\prime }\sim TN(h,\sigma_{h}^{2})I(0\leq h^{\prime }\leq h_{max})$. In applications we chose *σ_h _*to have acceptance ratios between 40% and 70%.

**• Updating allocations: **Using a Gibbs step, we draw the *n *allocations independently from

(8) $$\begin{array}{r} p(z_{i}=j|y,k,\mu ,\sigma^{2},x,h,\phi )\\ \propto \omega_{ij}N(y_{i}|\mu_{j},\sigma_{j}^{2})I[j\in \left\{1,\ldots k\right\}]. \end{array}$$

**• Updating *μ***, ***σ***^2^,**: **For each *j*, we consider the *k_max _*intervals and select that one with largest posterior probability, then sample *μ_j _*from a normal distribution truncated at this interval. In the iterations it may happen that two or more ${\mu^{\prime }}_{j}s$ are sampled to the same interval. In this case, we combine these components and update *k*. The new *μ_j_*, *σ_j_*, **x***_j_*, for the new formed component are taken to be the weighted sum of the original ones by the sample size. We then redefine **z **and calculate ***ω***. We draw $\sigma_{j}^{2}$ from its full conditional. See Appendix for the forms of the full conditionals.

For posterior inference, the primary parameters of interest are the weights ***ω*'**s. We propose an allocation rule as follows: at each iteration we record the probability of each SNP to belong to each of the *k_max _*components (we assign zero if a component is empty). After the MCMC is done, we average all the ***ω*'**s and assign a SNP to the component that has the largest probability. We check reproducibility of the clustering with different starting values.

The run-times of the various copy number algorithms can range from less than a minute to days depending on the algorithm and the probe density of the array platforms. When applied to newer high-density arrays almost all methods have relatively high run-times [17]. Reversible jump MCMC methods such as ours and RJaCGH [16] tend to be computationally expensive. Our current implementation may require several hours to > 1 day per chip. However, our current version is implemented in MatLab and we have not attempted to optimize the code. Programming in some version of C and parallel computing by chromosome and/or chromosome arm will likely significantly reduce the time.

## Results and Discussion

### Simulation Study

We first investigate the performance of our model through simulation experiments. In the next Section we compare our method with two alternative methods in the context of actual tumor samples from leukemia and ependymoma cancers.

We conducted two sets of simulations studies. The first set was designed to examine the influence of hyperparameters in the prior specifications: the scaling parameter, *ϕ*, of the logistic transformation for the GMRF and the number of smoothing neighbors, *nb*. Based on the results of the first set of experiments we then conducted a second set of experiments by setting these two parameters at fixed (default) values in order to assess performance of our algorithm. For scenarios with no contamination the log-ratios corresponding to copy number *j *were independently drawn from a normal distribution with the corresponding theoretical mean for copy number *j *and a standard deviation chosen to achieve a given SNR. For scenarios with contamination the log-ratios corresponding to copy number *j *were independently drawn from a normal distribution with mean $\log_{2}\frac{j(1-p)+2p+b}{2+b}$, where *p *is the percentage of contamination and *b *is the background factor, see [10].

In the first set of simulation studies we found that a small range of *ϕ *was suitable over different configurations. In particular, we investigated sensitivity by choosing different values in the range {.005, .01, .5, .1}. For the number of neighboring SNPs (on either side) over which to smooth in the GMRF the two values, 1 and 4 for of total of 2 or 8 neighbors for each SNP. Boundary SNPs at the ends of the chromosomes simply used fewer SNPs. Results and discussion from these sensitivity studies are reported in [Additional file 1]. Based on the results of the first set of experiments we then conducted a second set of experiments by setting these two parameters at fixed values, *ϕ *= 0.01 and *nb *= 4, and varying the signal-to-noise ratio and location of the copy number breakpoints. We also varied the number of SNPs constituting the aberration regions. In all simulations, the standard deviation (*σ_h_*) of the proposal distribution to update the smoothing parameter, *h*, was chosen so that acceptance ratios would be between 40% and 70%. For all cases reported we used 50, 000 sampling draws for inference after a 50, 000 iteration burn-in period.

For the second set of simulations we designed two patterns of copy number segments. For each pattern, we simulated four scenarios of SNP log-ratios. In practice, the log-ratios would be suitably normalized. The four scenarios are different configurations of true copy number, signal-to-noise ratio (SNR), normal cell contamination, and number of SNPs within in the CNA region. For each scenario we report misclassification, false-negative and false-positive rates. All rates in Tables 1, 2, 3 are based on 50 sample replicates.

**Table 1:** Misclassification rates from simulation study.

CN	# SNP	Scenario 1 .05/7.3/0	Scenario 2 .15/2.4/0
3	5	0	16
1	10	0	2
3	20	0	5
3	40	0	1

			

**CN**	**# SNP**	**Scenario 3** **.2/1.8/0**	**Scenario 4** **.2/1.5/20**

3	5	51	50
1	10	11	37
3	20	9	11
3	40	6	6

			

**CN**	**# SNP**	**Scenario 5** **.05/7.3/0**	**Scenario 6** **.15/2.4/0**

4	5	6	87
3	10	0	14
0	20	0	0
3	40	0	9

			

**CN**	**# SNP**	**Scenario 7** **.2/1.8/0**	**Scenario 8** **.2/1.5/20**

4	5	77	98
3	10	31	31
0	20	0	0
3	40	17	4

The entry is the misclassification rate, expressed as a percentage, over 50 replicates of one chromosome. Eight scenarios were simulated and defined by the given combination of true CN, number SNPs within the region of aberration, and SD/SNR/percent contamination. The SD and SNR are given on the log2 ratio scale under a true CN of 3. The true CN profile for Scenarios 1-4 is CN(#SNPs): 2(10), 3(5), 2(50), 1(10), 2(50), 3(20), 2(50), 3(40), 2(10). The true CN profile for Scenarios 5-8 is CN(#SNPs):2(10), 4(5), 2(50), 3(10), 2(50), 0(20), 2(50), 3(40), 2(10).

**Table 2:** False negative rates from simulation study.

CN	# SNP	Scenario 1 .05/7.3/0	Scenario 2 .15/2.4/0
3	5	0	16
1	10	0	2
3	20	0	5
3	40	0	1

			

**CN**	**# SNP**	**Scenario 3** **.2/1.8/0**	**Scenario 4** **.2/1.5/20**

3	5	47	50
1	10	11	37
3	20	5	11
3	40	3	6

			

**CN**	**# SNP**	**Scenario 5** **.05/7.3/0**	**Scenario 6** **.15/2.4/0**

4	5	0	0
3	10	0	7
0	20	0	0
3	40	0	1

			

**CN**	**# SNP**	**Scenario 7** **.2/1.8/0**	**Scenario 8** **.2/1.5/20**

4	5	4	6
3	10	20	31
0	20	0	0
3	40	4	4

The entry is the false-negative rate, expressed as a percentage, over 50 replicates of one chromosome.

See Table 1 for details.

**Table 3:** False positive rates from simulation study.

CN	# SNP	Scenario 1/5 .05/7.3/0	Scenario 2/6 .15/2.4/0
2	10	0	3
2	50	0	1

			

**CN**	**# SNP**	**Scenario 3/7** **.2/1.8/0**	**Scenario 4/8** **.2/1.5/20**

2	10	7	9
2	50	2	2

The entry is the false-positive rate, expressed as a percentage, over 50 replicates of one chromosome.

See Table 1 for details.

The *misclassification *rate reported is defined as *P *(*CN ≠ j *| *true CN *= *j*), for *j ≠ *2. For the special case *j *= 2 we obtain the *false-positive rate*, *FP *= *P *(*CN *≠ 2 | *true CN *= 2). The *false-negative rate *is defined as the chance of a true loss or gain classified as a normal copy number, *FN *= *P *(*CN *= 2 | *true CN *≠ 2).

We do not find it very useful to cite global rates since each depends on several factors, including the true CN, signal-to-noise ratio (SNR), normal cell contamination, and number of SNPs within the CNA region. We therefore report misclassification, false-negative and false positive rates given various combinations of these parameters. Other authors (e.g., [17]) define performance accuracy by breakpoint detection. This results in slightly different definitions of false-positive and false-negative rates than we do here. Since our model is based on mixture components corresponding to integer copy numbers it makes more sense for us to consider more specific false-negative and false-positive rates. As shown below, these rates also depend on factors other than true copy number.

A number of authors have used the simulation data of Willenbrock and Fridlyand [29] to assess their proposed copy number algorithms for aCGH data. Willenbrock and Fridlyand simulated CGH data using real breast cancer data. Their simulation parameters were deduced from the profiles of 145 breast tumor array CGH samples estimated with DNAcopy. For each sample, both the log-ratios (which emulate the aGCH data) and true copy number data are provided. One concern with these data is that they can be less noisy than real SNP data, since they emulate aCGH data. Since we are specifically interested in how well SNP data performs, we therefore chose to generate our own simulation data. We also note that our simulations generated simple text files of log-ratios. Therefore, we were unable to compare our method to those whose software implementation requires special data files, such as Affymetrix CEL files. Certain methods for CGH data, such as CBS, DNAcopy and GLAD, only require normalized data. However, these methods are for the three-state (gain, loss, normal) inference. In our simulation study we have regions of discrete copy numbers (0, 1, 2, 3, 4) so the results would not be comparable. The real data studies, however, did allow for such comparisons as we were able to obtain log-ratios from their analysis. In short, the simulations were for assessing our own method and the real data with validation were for performance assessment under real conditions and comparative purposes.

Table 1 shows misclassification rates (%) for eight different scenarios. Tables 2 and 3 show false-negative and false-positive rates, respectively. We first discuss the misclassification (MC) rates in Table 1.

**Figure 2:**
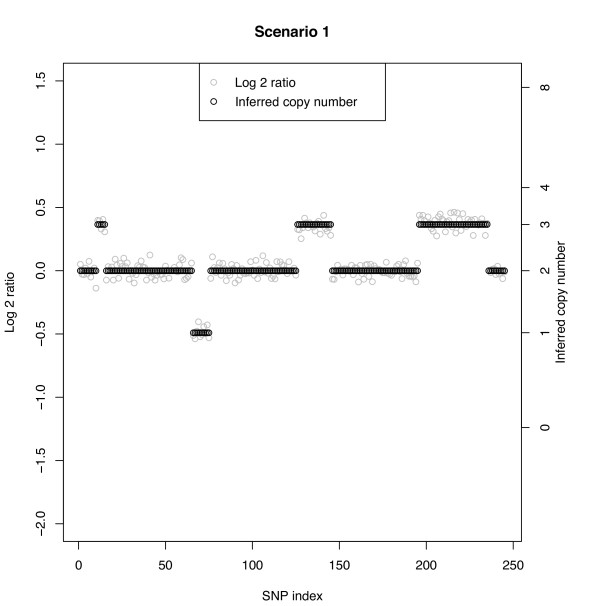
**Simulated data with Bayesian model CN inference**. Non-overlap scenario with SNR = 7.3 without normal cell contamination. The true CN profile is CN(#SNPs): 2(10), 3(5), 2(50), 1(10), 2(50), 3(20), 2(50), 3(40), 2(10).

**Figure 3:**
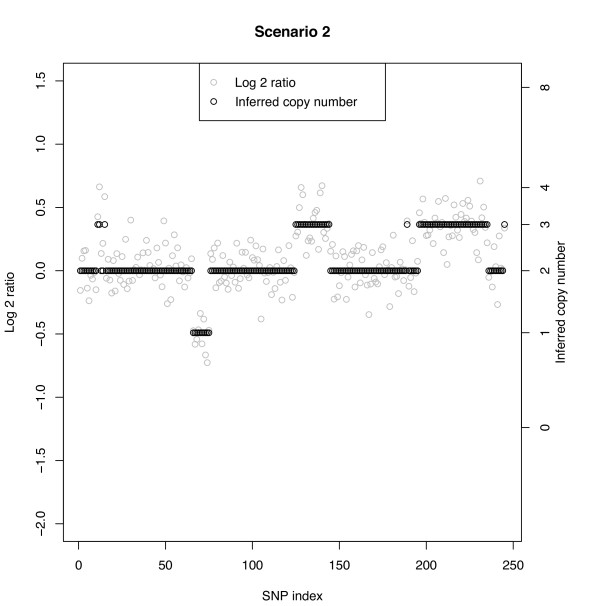
**Simulated data with Bayesian model CN inference**. SNR = 2.4 without normal cell contamination. The true CN profile is CN(#SNPs): 2(10), 3(5), 2(50), 1(10), 2(50), 3(20), 2(50), 3(40), 2(10).

**Figure 4:**
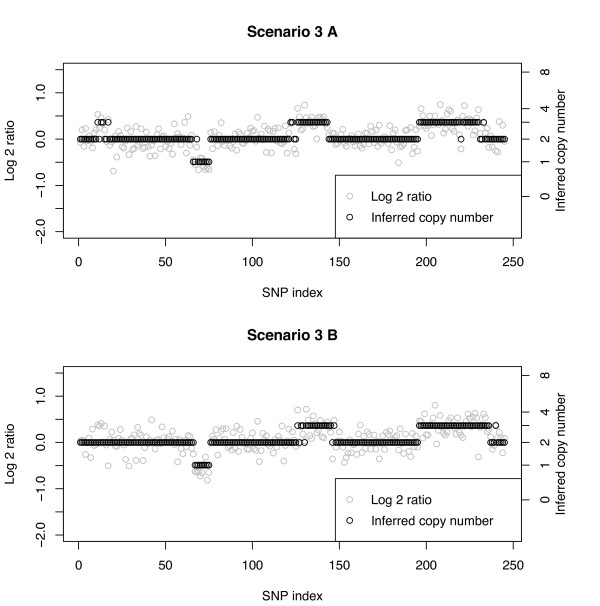
**Simulated data with Bayesian model CN inference**. SNR = 1.8 without normal cell contamination. Panels A and B are two possible observations under this scenario. At SNPs #11-15 the true CN = 3. (A) 1 of 5 SNPs classified as normal. (B) 5 of 5 SNPs classified as normal. The true CN profile is CN(#SNPs): 2(10), 3(5), 2(50), 1(10), 2(50), 3(20), 2(50), 3(40), 2(10).

**Scenarios 1-4: **These scenarios assume the following ordered copy number segments with number of SNPs given in parentheses: 2(10), 3(5), 2(50), 1(10), 2(50), 3(20), 2(50), 3(40), 2(10). The widths of the copy number segments (5, 10, 20, 40, 50) correspond to those considered by Rancoita et al. [20]. The SD and SNR are given on the log2 ratio scale under a true CN of 3. Since in this table we report misclassification rates, we do not show the segments corresponding to a true copy number of 2, which would be the false-positive rate (Table 3). The rows are ordered by segment as given above, excluding segments with a normal copy number. Figure 2 shows a typical data set under Scenario 1 in which the SNR of 7.3 leads to clearly non-overlapping log-ratios across the segments. In this case, the MC rate is 0% independent of CN aberration and number of SNPs defining the respective segments. Scenarios 2, 3, and 4 have increasingly smaller SNRs and for a given true CN aberration the MC rate increases with decreasing SNR (left to right across columns). Figure 3 shows a data set under Scenario 2 with a SNR of 2.4. The overlap between CN classes is mild, but clear change points can still be observed when there are at least 10 SNPs. Here, a few of the CN = 3 cases between SNPs 11-15 are classified as normals. Conversely, at about SNPs #190 and 250, normal CNs are classified as CN = 3. The largest MC rate (16%) under Scenario 2 is that corresponding to a segment with true CN = 3 and 5 SNPs. The other three cases under Scenario 2 with at least 10 SNPs have a MC rate of no more than 5%. Figures 4A and 4B show two data sets under Scenario 3 with a SNR under 2, namely SNR = 1.8. Figure 4A shows correct classification of 4 of 5 CN = 3 cases between SNPs 11-15, while Figure 4B shows all 5 of these CN = 3 cases misclassified as normals. However, Figure 4A shows more misclassifications of the CN = 3 cases between SNPs 230 and 240 than that in Figure 4B. With at least 10 SNPs in a segment, the MC rate is no more 11% under Scenario 3. Under Scenario 4 the SNR is 1.5 and as with Scenario 3 (SNR = 1.8) the MC rate is about 50% when only 5 SNPs define the segment. With a SNR as small as 1.5, a relatively large (> 10) number of SNPs are needed to accurately classify a copy number.

**Figure 5:**
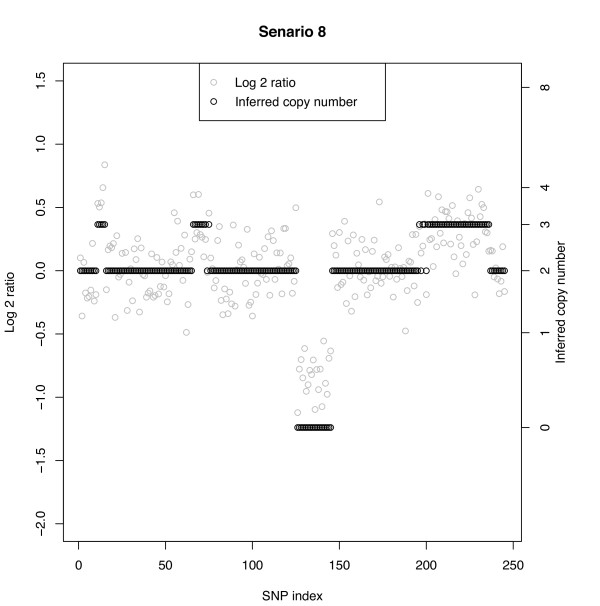
**Simulated data with Bayesian model CN inference**. SNR = 1.5 with 20% normal cell contamination. The true CN profile is CN(#SNPs): 2(10), 4(5), 2(50), 3(10), 2(50), 0(20), 2(50), 3(40), 2(10).

**Scenarios 5-8: **These represent the following ordered copy number segments with number of SNPs in parentheses: 2(10), 4(5), 2(50), 3(10), 2(50), 0(20), 2(50), 3(40), 2(10). As with Scenarios 1-4, for a given combination of CN and number of SNPs in the segment, the MC rate increases with decreasing SNR. Segments with a larger number of SNPs also lead to smaller MC rates than those with fewer SNPs. One interesting comparison is that between row 1 of Scenarios 1-4 (CN = 3 with 5 SNPs) with row 1 of Scenarios 5-8 (CN = 4 with 5 SNPs). Figure 5 shows a sample data set from Scenario 8 and there we observe that all five SNPs with CN = 4 at positions 11-15 are classified as CN = 3. Examining the misclassifications across all 50 replicates for this configuration we found that the vast majority of SNPs with CN = 4 were labelled as a 3; hence, the misclassification rate of 98%. Note that the false-negative rate for this situation (Table 2 row 1, Scenario 8) was only 6%. On the other hand, the MC rate under Scenario 4 with CN = 3 with 5 SNPs was 50%, approximately half that for CN = 4 in Scenario 8. In general, larger copy number aberrations are more difficult to correctly identify than smaller ones. Indeed, the log scale shrinks the larger copy number ratios toward smaller ones, leading to misclassifications. Line 3 shows MC rates under a true copy number of 0. Figure 5 shows how distinct this aberration is from its neighbors regardless of the size of the SNR; the MC is constantly 0%.

Table 2 shows false-negative rates. Except for minor differences, the false-negative rates for Scenarios 1-4 are the same as the broader misclassification rates (Table 1). This shows that most of the misclassifications in Scenarios 1-4 were losses and gains that were called normal. Where there are differences between Tables 1 and 2, we see that misclassification rates are at least as large as the false-negative rates as we would expect. It is worth noting that the aberrations studied in Scenarios 1-4 are neighbors of normal copy number, viz., CN = 3 is one additional copy and CN = 1 is one less copy. As such, it is not too surprising that the misclassification rates agreed with the false-negative rates. Especially in the presence of normal cell contamination we expect the log-ratios to regress toward the mean value of 0. This is contrast to Scenarios 5-8, which include more extreme aberrations of CN = 0 and CN = 4. Comparing the misclassification rates (Table 1) with the corresponding FN rates (Table 2), we see that the latter can be much smaller than the former. Large differences of MC vs FN rates are seen for CN = 4 in Scenarios 6 (87% vs 0%), 7 (77% vs 4%), and 8 (98% vs 6%). Taken together this implies that almost all of the misclassifications for CN = 4 were called as CN = 3 and very few as CN = 2. A manual calculation of the calls confirms this conclusion. Smaller differences between MC and FN rates occur in Scenario 6 with CN = 3 and 10 SNPs (line 2, Tables 1 and 2); the MC rate is 14% and the FN rate is 7%. Here, half of the 14% is due to normal calls and the other half to calls of CN = 4. In Scenario 7 with true CN = 3 and 10 SNPs the MC rate of 31% is 20% CN = 2 (false-negative) and 11% CN = 4. Similarly, the MC rates of 9% and 17% for Scenarios 6 and 7 with CN = 3 and 40 SNPs (line 4, Tables 1 and 2), respectively, are only due to false calls of CN = 2 and CN = 4. It is, therefore, seen that when a true copy number of 3 is misclassified, it tends to be called a CN = 4 with a smaller percentage of normal calls, CN = 2. And, as discussed above, a true CN = 4 tends to be called a 3 when misclassified. In this sense, if an investigator is only calling loss/normal/gain, even though misclassifications occur under true copy numbers of 3 and 4, they would both be correctly called as gains with a small percentage of CN = 2 (false-negative) calls. This is at least the behavior of the Bayes mixture model; other methods may apportion the misclassifications differently. In all scenarios (1-8) we observe a misclassification rate and a false-negative rate of 0% for CN = 0 and 20 SNPs. No matter the signal-to-noise ratio, the distribution of log-ratios for CN = 0 is well separated from the other copy number distributions and its call is constantly correct. For CN = 1, the misclassification rates and corresponding false-negative rates are equal, showing that when misclassified this copy number is called a normal (false-negative). Table 3 shows false-positive (FP) rates defined as a true normal copy number being classified as a gain or loss: *P*(*CN *≠ 2 | *CN *= 2). Since the two patterns of copy number structure differed only in their gain and loss patterns we combined the data for the normal copy number segments. Thus the FP rates are based on 100 replicates instead of 50 as with the MC and FN rates in Table 1. As with the FN rate, for a fixed number of SNPs defining the normal segment, the FP positive rate increases with decreasing SNR. And, for a given combination of SNR and normal cell contamination, the FP rate decreases with an increasing number of SNPs in the segment. Under the most difficult configuration considered, 10 SNPs with a SNR of 1.5 and 20% contamination, the false-positive rate was only 9%.

Rancoita et al. [20] compared their mBPCR method with six other methods and found that in general no method, including their own, was able to detect aberrations of width 5-10 probes. Lai et al. [30] reached similar conclusions. Use of alternative estimators for a certain covariance parameter led to the detection of these smaller segments, but this was accompanied by dividing larger segments into sub-segments. Our method, too, had trouble with regions defined by only 5 probes, although regions with 10 probes were fairly well identified unless the signal-to-noise ratio was on the order of 1.5.

### Real Data Application

To further assess the Bayes mixture model, we analyzed Affymetrix 250*K *array data from cancer patients who had either leukemia or ependymoma. Data were obtained from Texas Children's Hospital, Houston, TX. In addition to comparing our results with the popular CNAG software, we also provide biological validation. Some of these cases, in fact, had karyotyping and FISH data for validation. Others were validated using quantitative PCR on selected regions and aCGH data. We also briefly comment on comparisons with some results from the PennCNV algorithm.

One important feature of the CNAG software used to estimate copy number is the fact that it adjusts the observed log-ratios for variation in GC content across the probes. Integer copy numbers are subsequently inferred from the GC adjusted log-ratios using a hidden Markov model. To make comparable comparisons between the Bayes and the CNAG methods we applied the Bayes model to the GC adjusted log-ratios from CNAG. One relatively recent issue arising in the analysis copy number aberration detection is the so-called "genome wave" [11,12], a genome-wide spatial autocorrelation pattern in signal intensity data that may be confounded with the copy number profile across a chromosome. As a result the genome wave may lead to inflated false-positive rates in copy number calls. The genome wave has been consistently detected in both CGH and SNP based platforms. Diskin et al. [12] and the references therein describe possible genomic features underlying the wave effect and pre-processing methods to remove the wave effect prior to the analysis of copy number. It has been fairly well established that an adjustment for GC content largely removes the wave effect from the signal intensities [12]. Since we are using GC adjusted log-ratios from CNAG for the real data application we did not expect to observe a wave effect in our data and indeed none was present as shown in [Additional file 1].

**Figure 6:**
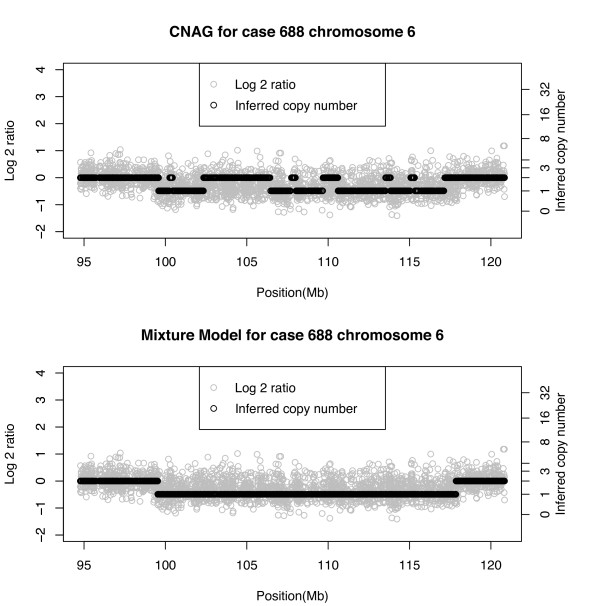
**Real data, case 688, chromosome 6**. Comparison of CNAG and Bayesian mixture model copy number inference.

Figure 6 shows normalized log-ratios by their genomic location over a segment of chromosome 6 from an aneuploidy case (#688). Black circles are corresponding inferred integer copy numbers. For this case FISH data suggest a loss of 6q12-6q21(63.4 - 114.6 Mb, hg19), a 51.2 Mb long region. The top panel shows results from CNAG and the bottom panel shows results from the Bayesian model. Both methods suggest that the loss is actually smaller than 51.2 Mb, ranging from approximately 99-118 Mb, a 19 Mb region. The SNP based analysis appears to have captured the boundaries of loss regions more precisely as we would expect given its higher resolution than cytogenetics. The Bayesian method gives smoother results as indicated by the longer stretches of the same inferred copy number, whereas the CNAG method varies more in the inferred copy number over regions of loss. The Bayesian results coincide better with what is expected in practice in that copy number aberrations usually occur in contiguous regions within a chromosome; the CNAG results seem to indicate unstable results rather than real structural changes. In particular, note the region from approximately 102:5 *Mb *to 107 *Mb*, covering 5:5 *Mb*, which is assigned copy number 2 by CNAG. Although the reason for the misclassification is unknown, it would appear that it is not due to a small number of SNP loci in the region. Both methods appear to agree on the first change point at 99 Mb in going from a normal region to a gain of CN = 3. At the other end, it may be that the Bayes method ends the loss region at 188 Mb, while the CNAG boundary is at 117 MB; however, a formal validation would be needed to discriminate this apparent difference.

**Figure 7:**
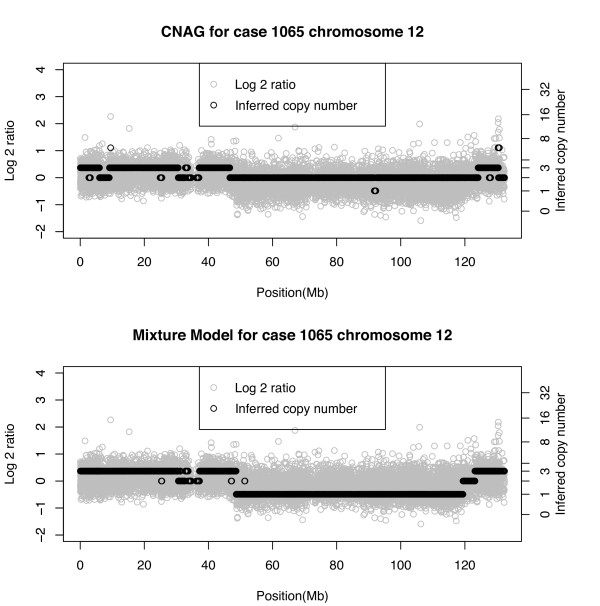
**Real data, case 1065, chromosome 12**. Comparison of CNAG and Bayesian mixture model copy number inference.

**Figure 8:**
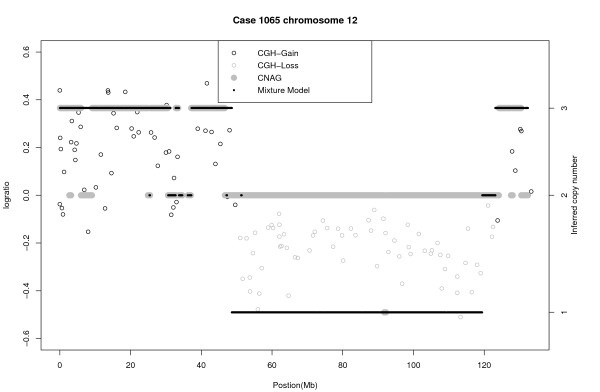
**Real data, case 1065, chromosome 12**. Comparison of CNAG, Bayesian mixture model, and aCGH GLAD copy number inference.

In the next example we consider an ependymoma case (#1065). Figure 7 shows chromosome 12 for this case. Both methods infer CN = 3 for much of the region from 0 to 30 Mb, although prior to the centromere located at 35 Mb there is a signal for CN = 2. The main difference between the two algorithms centers on the segment from approximately 45 to 120 Mb. This segment is identified as CN = 2 by CNAG and CN = 1 by the Bayes algorithm. In order to validate this result we performed qPCR on two regions in this stretch: at approximately 55 Mb, and 110 Mb. The qPCR average (95% confidence interval) copy numbers for these two regions were 1.43 (1.2, 1.71) and 1.55 (1.33, 1.81), respectively. These validation results support the loss identified by the Bayes method. Given that the distribution of the log-ratios from 45-120 Mb agrees with those at 55 Mb and 110 Mb we conclude that the Bayes method has correctly identified a loss from 45-120 Mb. There is also a discrepancy at the end of the chromosome. The Bayes method infers a contiguous gain of CN = 3 (125-134 Mb), whereas CNAG has a gain (123-130 Mb) followed by a normal region (130-134 Mb). qPCR validation was performed at locations 127 Mb and 132 Mb and we found means of (95% confidence interval) of 2.81 (2.48, 3.18) and 2.36 (1.96, 2.85), respectively. The first qPCR result suggests a CN of 3. The second result is somewhat less conclusive as CN = 2 is just inside the confidence interval, which is approximate. To further validate this case, we therefore generated aGCH data via an aCGH platform with 2,621 BACs and a resolution of 3 Mb. Having analyzed the aCGH data with the GLAD software from *Bioconductor *http://www.bioconductor.org/, Figure 8 shows the aCGH results overlayed by the Bayes and CNAG results. The aCGH data show a gain of CN = 3 from the beginning of the chromosome to the centromere at 35 Mb. With the exception of a few small regions both the Bayes and CNAG methods agree with GLAD in this region. From the beginning of the *q *arm to 45 Mb all three methods show CN = 3. From 45 Mb to 120 Mb, GLAD clearly indicates a loss, and from 120 Mb to the end at 134 Mb a gain. It thus appears that the Bayes method correctly identified a gain at the end of the chromosome, whereas CNAG inferred a normal copy number.

**Figure 9:**
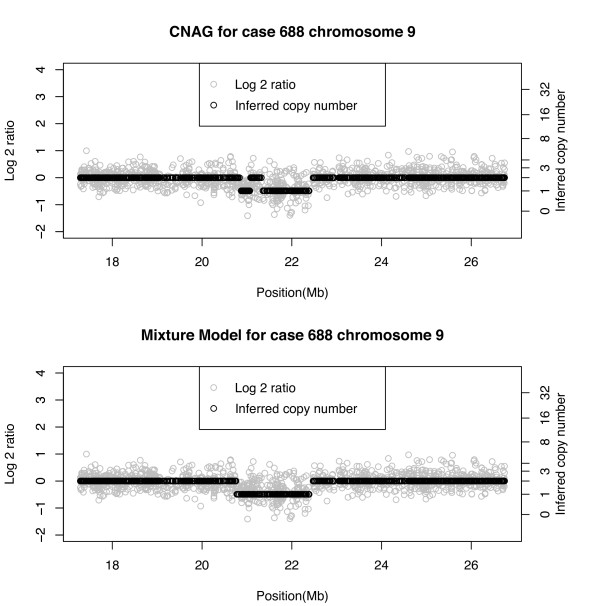
**Real data, case 688, chromosome 9**. Comparison of CNAG and Bayesian mixture model copy number inference.

In the two real cases (688 and 1065) discussed thus far, the Bayesian method showed regions of loss, a 19 Mb region in case 688 (Figure 5) and a 75 Mb region in case 1065 (Figure 6). The mean log2-ratio of the loss region in case 688 was -0.3 and in case 1065 it was -0.18. Although it is not obvious from Figure 6, the difference in means and the fact that both corresponded to CN = 1 indicate that the EPN 1065 case had a tumor sample with a relatively high degree of normal cell contamination. This may be why CNAG inferred the large 75 Mb region as a normal region. Below in Figure 9 we show case 688, chromosome 9 in which a much smaller region of size 1.5 Mb is detected as a loss. FISH validation confirms this as a CN = 1 loss. As in the previous figures, CNAG is as not as stable as the Bayesian algorithm in estimating the same copy number over a contiguous aberration.

We performed additional comparisons with the PennCNV algorithm. For the chromosome 6 region from case 688, the region from about 99 Mb to 118 Mb was identified as copy number 1 by our mixture model, while PennCNV only detected four very short regions inside the region we identified. For the chromosome 12 region from case 1065, the region 45-120 Mb is identified as a loss by our method while PennCNV gives 2 very noisy results with most part of this region identified as CN = 2. Also, as we validated, the tail region of this chromosome is a gain, while PennCNV does not detect it. Finally, for the chromosome 9 region from case 688, our mixture model detected the region from about 20.5 Mb to 22.5 Mb as copy number 1, while PennCNV only detected a small part of it (from 20.5-21 Mb). We report plots from the PennCNV algorithm in the [Additional file 1].

## Conclusions

The array-based comparative genomic hybridization microarray is a widely accepted method for estimating genomic copy number. As the CGH BACs are relatively large segments, the CGH estimates tend to be robust. On the other hand, the large segments do not allow detection of small CNVs. The SNP genotyping array provides an alternative to CGH, which is expected to identify genomic alterations with a higher resolution. Most SNP array algorithms use a hidden Markov model to infer integer copy numbers, and the component means tend to be set at the theoretical values. However, due to normal cell contamination, which occurs in most tumor samples, log-ratios can be shrunk toward zero, indicating a normal copy number. Consequently, in the presence of a high percentage of contamination, losses or gains may not be detectable. As of this writing, we are not aware of existing algorithms that account for this problem.

We have developed a Bayesian spatial normal mixture model to estimate copy number for SNP array platforms where the means of the components accommodate cell contamination. By using neighboring copy number information on either side of each SNP locus we can generate smoother maps than those based on HMMs. We have shown with a simulation study that our algorithm can detect both long and short segments quite precisely. Our results do not show sensitivity to different values of the scaling factor *ϕ *in the prior distribution and to the number of neighbors as long as *ϕ *is chosen to be small enough. By applying our method to real cancer data, we have demonstrated that our algorithm can do as well as CNAG, a very popular and accurate algorithm used with SNP arrays, and in certain cases performs better. In addition, our algorithm provides smoother realizations than CNAG. The Bayesian mixture model could be extended in a few ways. To more precisely smooth over neighboring probes, it would be helpful to account for inter-probe distance perhaps as a weighting factor when averaging neighboring information. The log-ratio copy number means could also be included as parameters with priors reflecting knowledge of normal cell contamination.

## Competing interests

The authors declare that they have no competing interests.

## Authors' contributions

CL and RG developed the original concept of the study. BG, AV, MV and RG designed the model, performed the analyses and drafted the manuscript. JW, TKM and CL designed the experimental studies, collected the data, contributed to the interpretation of the results and critically revised the manuscript. All authors read and approved the final manuscript.

## Appendix

We report here full details of the derivations for the MCMC algorithm.

**1**. Updating *k*: According to Richardson and Green (1997), a new component is accepted with probability *min*{1,α} $\alpha =\frac{p(\theta^{\prime }|y)r_{m}(\theta^{\prime })}{p(\theta |y)r_{m}(\theta )q(u)}|\frac{\partial \theta^{\prime }}{\partial (\theta ,u)}|$. In our *θ *= (*k*, ***μ***, **σ**^2^, **x**). We have the following distributions

$$\begin{array}{lll} \frac{r_{m}(\theta^{\prime })}{r_{m}(\theta )q(u)} & = & \frac{(1-b_{k+1})\frac{1}{k+1}}{b_{k}\frac{1}{k_{max}-k}p(\mu_{*})p(\sigma_{*}^{2})p(x_{*})}\\ p(\theta^{\prime }|y) & \propto & \left(\prod_{i=1}^{n}\sum_{j=1}^{k+1}{\omega^{\prime }}_{ij}N(y_{i}|{\mu^{\prime }}_{j},{\sigma^{\prime }}_{j}^{2})\right)\\ & & \times \left(\prod_{j=1}^{k+1}p({\mu^{\prime }}_{j})\right)\left(\prod_{j=1}^{k+1}p({\sigma^{\prime }}_{j}^{2})\right)\\ & & \times \left(\prod_{j=1}^{k+1}p({x^{\prime }}_{j})\right)p(k+1)\\ p(\theta^{\prime }|y) & \propto & \left(\prod_{i=1}^{n}\sum_{j=1}^{k}\omega_{ij}N(y_{i}|{\mu^{\prime }}_{j},\sigma_{j}^{2})\right)\\ & & \times \left(\prod_{j=1}^{k}p(\mu_{j})\right)\left(\prod_{j=1}^{k}p(\sigma_{j}^{2})\right)\\ & & \times \left(\prod_{j=1}^{k}p(x_{j})\right)p(k) \end{array}$$

Since we add a component to the original vector by an identity transformation our Jacobian is equal to 1. We therefore have

α = (1−bk+1)1k+1p(k+1)bk1kmax−kp(k)×∏i=1n∑j=1k+1ω′ijN(yi | μ′j,σ′j2)∑j=1kωijN(yi | μj,σj2)

which gives (6). Similar derivations hold for (7).

**2**: Updating **x**: For each location *i*, the full conditional of (*x*_*i*1_,..., *x_ik_*) is

$$\begin{array}{c} \sum_{j=1}^{k}\omega_{ij}N(y_{i}|\mu_{j}\text{,}\sigma_{j}^{\text{2}})\\ \times \prod_{j=1}^{k}N\left(x_{ij}|\frac{h\Sigma_{i^{\prime }\sim i}x_{i^{\prime }j}}{1+hn_{i}},\frac{1}{1+hn_{i}}\right) \end{array}$$

where *n_i _*is the number of neighbors at location *i*. We therefore use a proposal distribution of the type

$$\prod_{j=1}^{k}N\left({x^{\prime }}_{ij}|\frac{h\sum_{i^{\prime }\sim i}x_{i^{\prime }j}}{1+hn_{i}},\frac{1}{1+hn_{i}}\right).$$

**3**: **Updating ***h*: The full conditional for *h *is

$$\begin{array}{c} c{\left(h\right)}^{k}exp\left(-\frac{h}{2}\sum_{j=1}^{k}{\sum_{{}_{i\sim i^{\prime }}}\left(x_{ij}-x_{i^{\prime }j}\right)}^{2}\right)\\ \times I(0\leq h\leq h_{max}). \end{array}$$

We use a Metropolis-Hastings random walk with proposal a truncated normal distribution, $h^{\prime }\sim TN(h,\sigma_{h}^{2})I(0\leq h^{\prime }\leq h_{max})$. The acceptance probability is given by,

$$\begin{array}{c} min(1,\frac{c{\left(h^{\prime }\right)}^{k}\left(\Phi (\frac{h_{max}-h}{\sigma_{h}})-\Phi (\frac{-h}{\sigma_{h}})\right)}{c{\left(h\right)}^{k}\left(\Phi (\frac{h_{\text{max}}-h^{\prime }}{\sigma_{h}})-\Phi (\frac{-h^{\prime }}{\sigma_{h}})\right)}\\ \times \frac{exp\{-\frac{h^{\prime }}{2}\sum_{j=1}^{k}\sum_{{}_{i\sim i^{\prime }}}{\left(x_{ij}-x_{i^{\prime }j}\right)}^{2}\}}{exp\{-\frac{h}{2}\sum_{j=1}^{k}\sum_{i\sim i^{\prime }}{\left(x_{ij}-x_{i^{\prime }j}\right)}^{2}\}}) \end{array}$$

**4: Updating *μ*, *σ*^2^**: The full conditional for (*μ*_1_, ...,*μ_k_*) is

$$\prod_{j=1}^{k}N\left(\frac{\sum_{i:z_{i}=j}y_{i}}{N_{j}},\;\frac{\sigma_{\text{j}}^{\text{2}}}{N_{j}}\right)I(a_{j}\text{<}\mu_{\text{j}}\text{<}b_{j})$$

where *N_j _*is the number of SNPs that are assigned to component *j*. For each *j*, we consider the *k_max _*intervals and select that one with largest posterior probability, then sample *μ_j _*from a normal distribution truncated at this interval. The full conditional for $\sigma_{j}^{2}$ is

$$\sigma_{j}^{2}\sim Inverse-Gamma\;(A,B)$$

with $A=\frac{1}{2}N_{j}+\alpha_{\sigma^{2}}$ and $B=\frac{1}{2}\sum_{i:z_{i}=j}{\left(y_{i}-\mu_{j}\right)}^{2}+\beta_{\sigma^{2}}$.

## Supplementary Material

Additional file 1 **Supplementary File**. Results on a first set of simulated data. Plots on absence of genomic wave. Results from the PennCNV algorithm. Click here for file
