# Supplementary material for: Bayesian estimation of genomic copy number with single nucleotide polymorphism genotyping arrays
Source: BMC Res Notes. 2010 Dec 30;3:350. doi: 10.1186/1756-0500-3-350 (PMC3023756; doi:10.1186/1756-0500-3-350)
Supplement: Additional file 1 — Supplementary File. Results on a first set of simulated data. Plots on absence of genomic wave. Results from the PennCNV algorithm. [file 1756-0500-3-350-S1.PDF]

Supplementary File

October 21, 2010

1 First set of simulation studies

We simulated 8 data sets representing 8 sets of (normalized) SNP log-ratios. Four data sets represent the case where there is a clear separation among three contiguous segments whose ordered copy numbers are 2, 4, and 2, corresponding to normal/gain/normal. We call this scenario the "non-overlap" case. Figure 1 shows four versions of this case. In each panel there are 100 SNPs to the left of the gain segment and 100 SNPs to the right. The four versions differ by how many SNPs are in the gain segment: 100, 50, 25, 10. The second set of four data sets are analogous to the first set except for the fact that the log-ratios of the gain segment are not well separated from the normal (CN=2) log-ratios on either side. We call this case the "overlap" case. Figure 2 shows representative samples corresponding to the gain segment having 100, 50, 25, and 10 SNPs. In all cases examined we computed misclassification numbers and false negative fractions (i.e., numbers of false negatives divided by the numbers of non-normal SNPs). We comment on some of the results below. Figures and tables for the full simulation study are available upon request.

Non-overlap Case

In the non-overlap scenario the log-ratios corresponding to CN=2 SNPs were independently drawn from a $N(0, .1^2)$ distribution. The log-ratios corresponding to CN=4 SNPs were drawn from a $N(.6, .1^2)$, for all four SNP sample sizes of 10, 25, 50, and 100. For this case we obtained excellent results and observed no sensitivity to the parameter ϕ and to the number of neighbors. We show a representative result in Figure 1. False negative fractions are given in Table 1 and number of misclassifications for the 50 and 10 gain cases are given in Tables 2 and 3.

Overlap Case

In the overlap case the log-ratios of the normal copy number SNPs were sampled from a $N(0, .15^2)$, and the gains from a $N(.3, .15^2)$. For this case we obtained excellent results in all cases except one. When $\phi = .1$ the algorithm can detect the three cases

with 100/50/25 SNPs in the middle, but cannot detect the short segment of 10 SNPs, see Figures 2 and 3 and Tables 1,2 and 3.

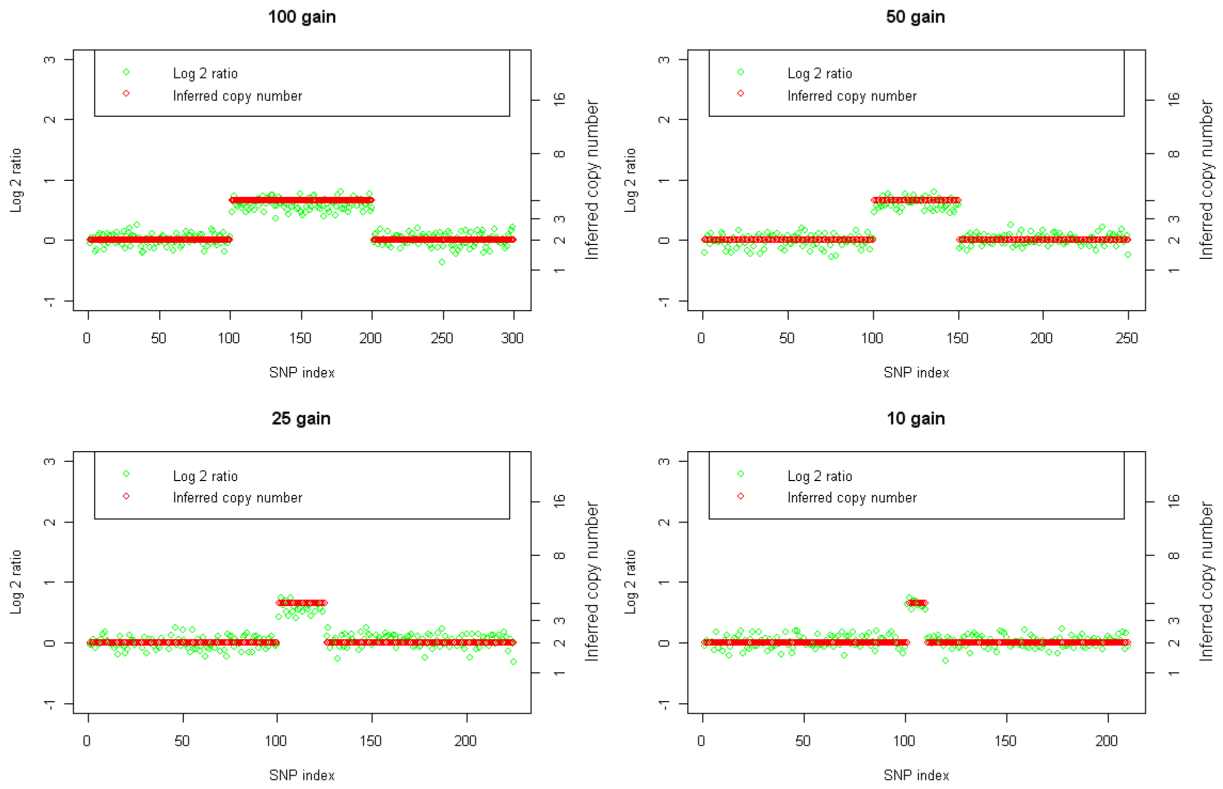

Figure 1: Simulated data: Non-overlap cases with $\phi = .1$ and 1 neighbor each side

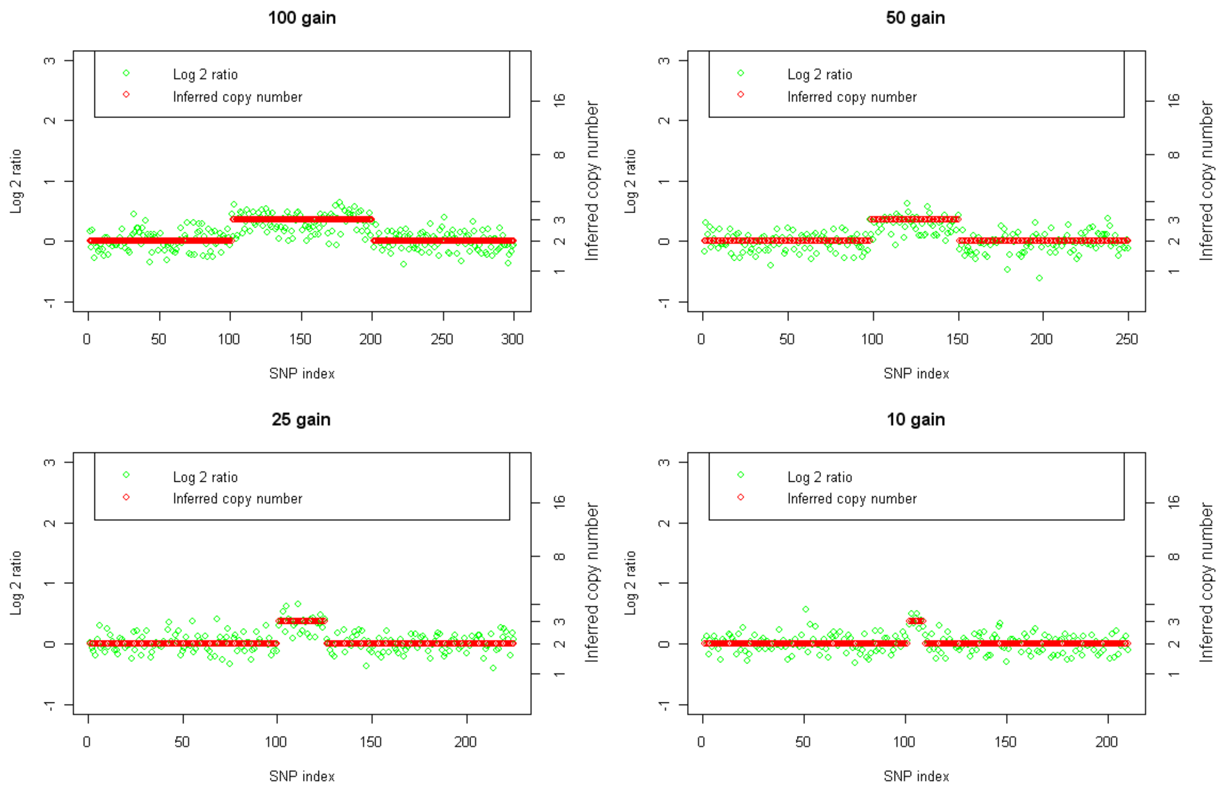

Figure 2: Simulated data: Overlap cases with $\phi = .05$ and 1 neighbor each side

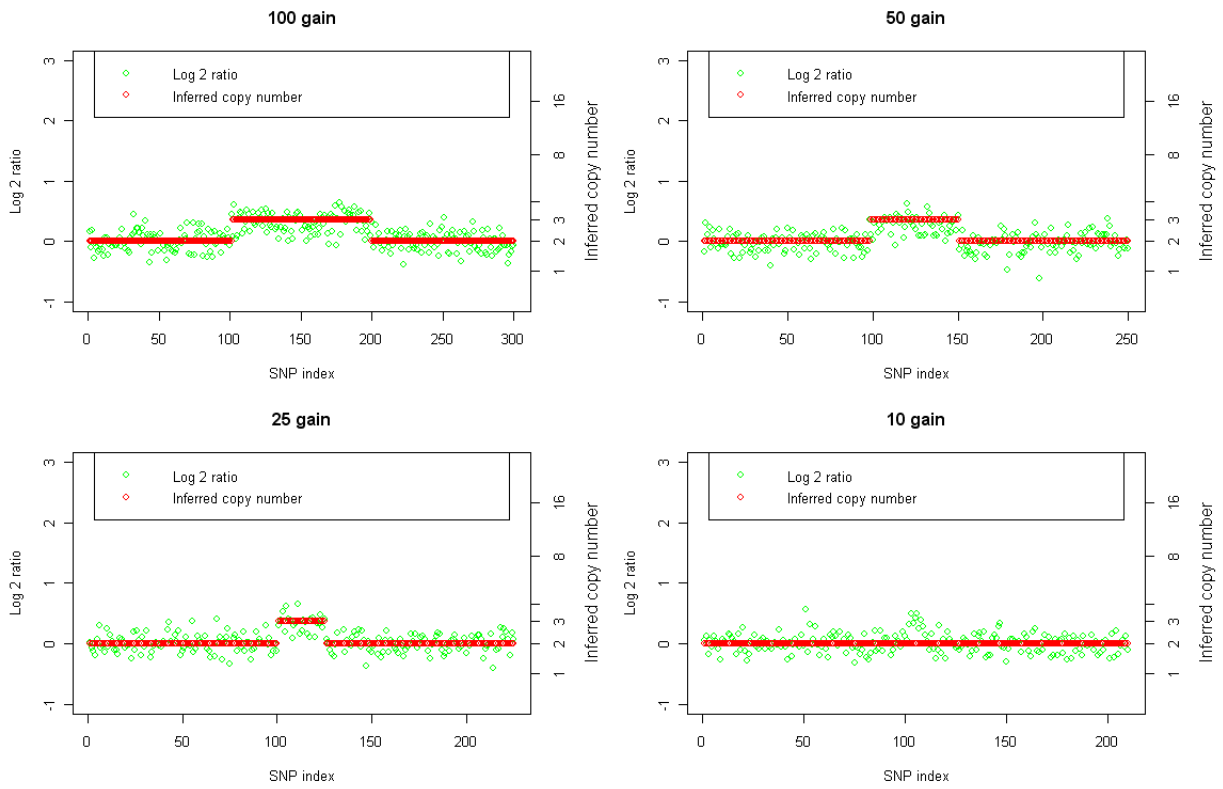

Figure 3: Simulated data: Overlap cases with $\phi = .1$ and 1 neighbor each side

Tables

Table 1 - Simulation study: False negative for all cases examined

**False negative fraction for 100 gain**									
	$\phi = .005$		$\phi = .01$		$\phi = .5$		$\phi = .1$		
nb	1	4	1	4	1	4	1	4	avg
overlap	0	.02	.01	.02	.01	.02	.02	.02	.015
non-overlap	0	0	0	0	0	0	0	0	0
avg	0	.01	.005	.01	.005	.01	.01	.01	.0075

**False negative fraction for 50 gain**									
	$\phi = .005$		$\phi = .01$		$\phi = .5$		$\phi = .1$		
nb	1	4	1	4	1	4	1	4	avg
overlap	0	0	0	0	0	0	0	0	0
non-overlap	0	0	0	0	0	0	0	0	0
avg	0	0	0	0	0	0	0	0	0

**False negative fraction for 25 gain**									
	$\phi = .005$		$\phi = .01$		$\phi = .5$		$\phi = .1$		
nb	1	4	1	4	1	4	1	4	avg
overlap	0	0	0	0	0	0	0	0	0
non-overlap	0	0	0	0	0	0	0	0	0
avg	0	0	0	0	0	0	0	0	0

**False negative fraction for 10 gain**									
	$\phi = .005$		$\phi = .01$		$\phi = .5$		$\phi = .1$		
nb	1	4	1	4	1	4	1	4	avg
overlap	.4	.2	.2	.2	.2	.2	1	1	.425
non-overlap	0	0	0	0	0	0	.1	.2	.038
avg	.2	.1	.1	.1	.1	.1	.55	.6	.232

Table 2 - Simulated data: Miss-classification numbers for the 50 gain cases

$50\ gain\ non-overlap$

	$\phi = .005$, $nb = 1$		$\phi = .005$, $nb = 4$		$\phi = .01$, $nb = 1$		$\phi = .01$, $nb = 4$	
Truth	$cn = 2$	$cn = 4$	$cn = 2$	$cn = 4$	$cn = 2$	$cn = 4$	$cn = 2$	$cn = 4$
$cn = 2$	200	0	200	0	200	0	200	0
$cn = 4$	0	50	0	50	0	50	0	50

	$\phi = .05$, $nb = 1$		$\phi = .05$, $nb = 4$		$\phi = .1$, $nb = 1$		$\phi = .1$, $nb = 4$	
Truth	$cn = 2$	$cn = 4$	$cn = 2$	$cn = 4$	$cn = 2$	$cn = 4$	$cn = 2$	$cn = 4$
$cn = 2$	200	0	200	0	200	0	200	0
$cn = 4$	0	50	0	50	0	50	0	50

$50\ gain\ overlap$

	$\phi = .005$, $nb = 1$		$\phi = .005$, $nb = 4$		$\phi = .01$, $nb = 1$		$\phi = .01$, $nb = 4$	
Truth	$cn = 2$	$cn = 3$	$cn = 2$	$cn = 3$	$cn = 2$	$cn = 3$	$cn = 2$	$cn = 3$
$cn = 2$	197	3	198	2	198	2	198	2
$cn = 3$	0	50	0	50	0	50	0	50

	$\phi = .05$, $nb = 1$		$\phi = .05$, $nb = 4$		$\phi = .1$, $nb = 1$		$\phi = .1$, $nb = 4$	
Truth	$cn = 2$	$cn = 3$	$cn = 2$	$cn = 3$	$cn = 2$	$cn = 3$	$cn = 2$	$cn = 3$
$cn = 2$	198	2	197	3	198	2	197	3
$cn = 3$	0	50	0	50	0	50	0	50

Table 3 - Simulated data: Miss-classification numbers for the 10 gain cases

10 *gain non − overlap*

	$\phi = .005$, $nb = 1$		$\phi = .005$, $nb = 4$		$\phi = .01$, $nb = 1$		$\phi = .01$, $nb = 4$	
Truth	$cn = 2$	$cn = 4$	$cn = 2$	$cn = 4$	$cn = 2$	$cn = 4$	$cn = 2$	$cn = 4$
$cn = 2$	200	0	200	0	200	0	200	0
$cn = 4$	0	10	0	10	0	10	0	10

	$\phi = .05$, $nb = 1$		$\phi = .05$, $nb = 4$		$\phi = .1$, $nb = 1$		$\phi = .1$, $nb = 4$	
Truth	$cn = 2$	$cn = 4$	$cn = 2$	$cn = 4$	$cn = 2$	$cn = 4$	$cn = 2$	$cn = 4$
$cn = 2$	200	0	200	0	200	0	200	0
$cn = 4$	0	10	0	10	1	9	2	8

10 *gain overlap*

	$\phi = .005$, $nb = 1$		$\phi = .005$, $nb = 4$		$\phi = .01$, $nb = 1$		$\phi = .01$, $nb = 4$	
Truth	$cn = 2$	$cn = 3$	$cn = 2$	$cn = 3$	$cn = 2$	$cn = 3$	$cn = 2$	$cn = 3$
$cn = 2$	200	0	200	0	200	0	200	0
$cn = 3$	4	6	2	8	2	8	2	8

	$\phi = .05$, $nb = 1$		$\phi = .05$, $nb = 4$		$\phi = .1$, $nb = 1$		$\phi = .1$, $nb = 4$	
Truth	$cn = 2$	$cn = 3$	$cn = 2$	$cn = 3$	$cn = 2$	$cn = 3$	$cn = 2$	$cn = 3$
$cn = 2$	200	0	200	0	200	0	200	0
$cn = 3$	2	8	2	8	10	0	10	0

2 Genomic Wave

In each of the following three plots, green dots are logratios. The red line is the loewss curve with window size .3.

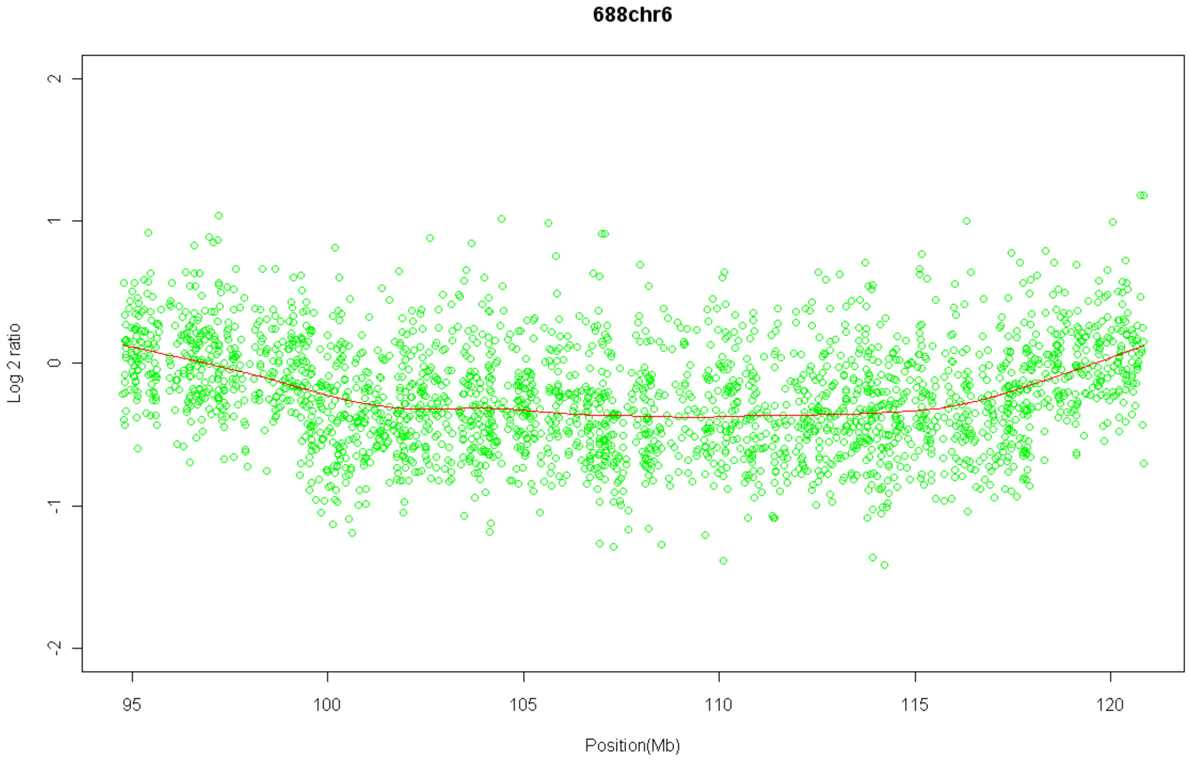

Figure 4: Case 688 chromosome 6

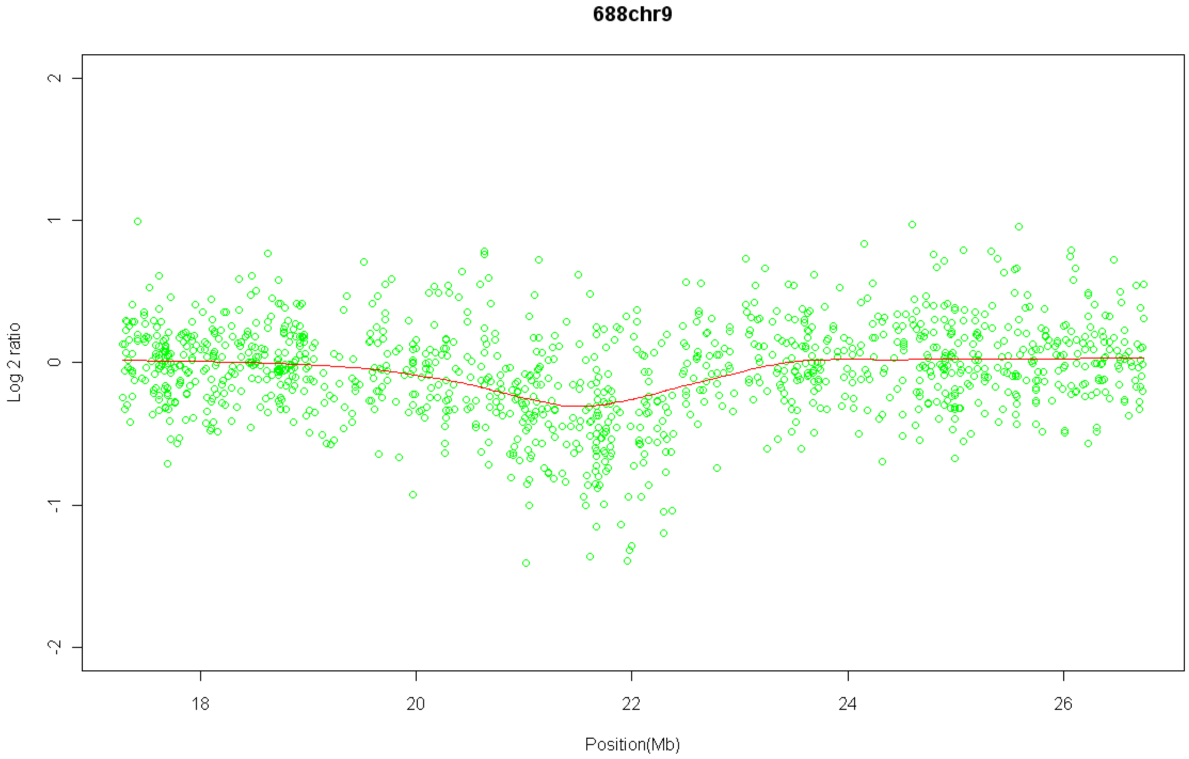

Figure 5: Case 688 chromosome 9

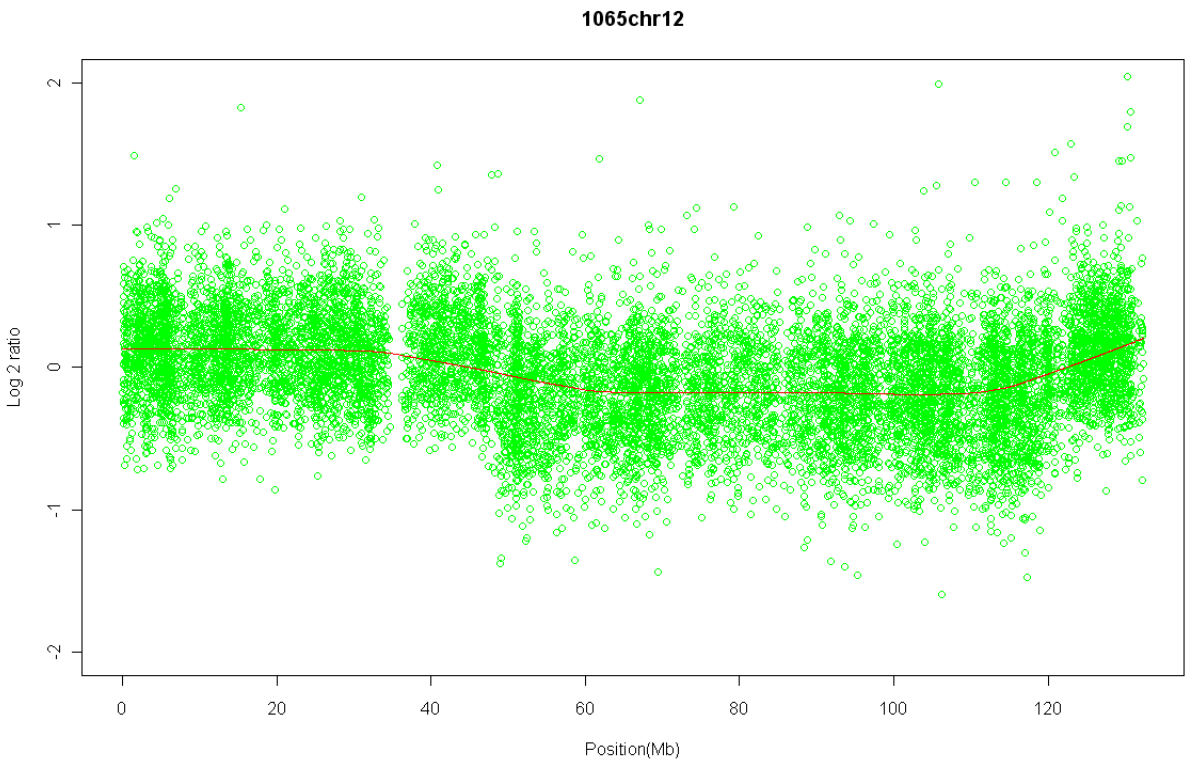

Figure 6: Case 1065 chromosome 12

3 PennCNV

In the following two plots, black dots are inferred copy numbers from PennCNV.

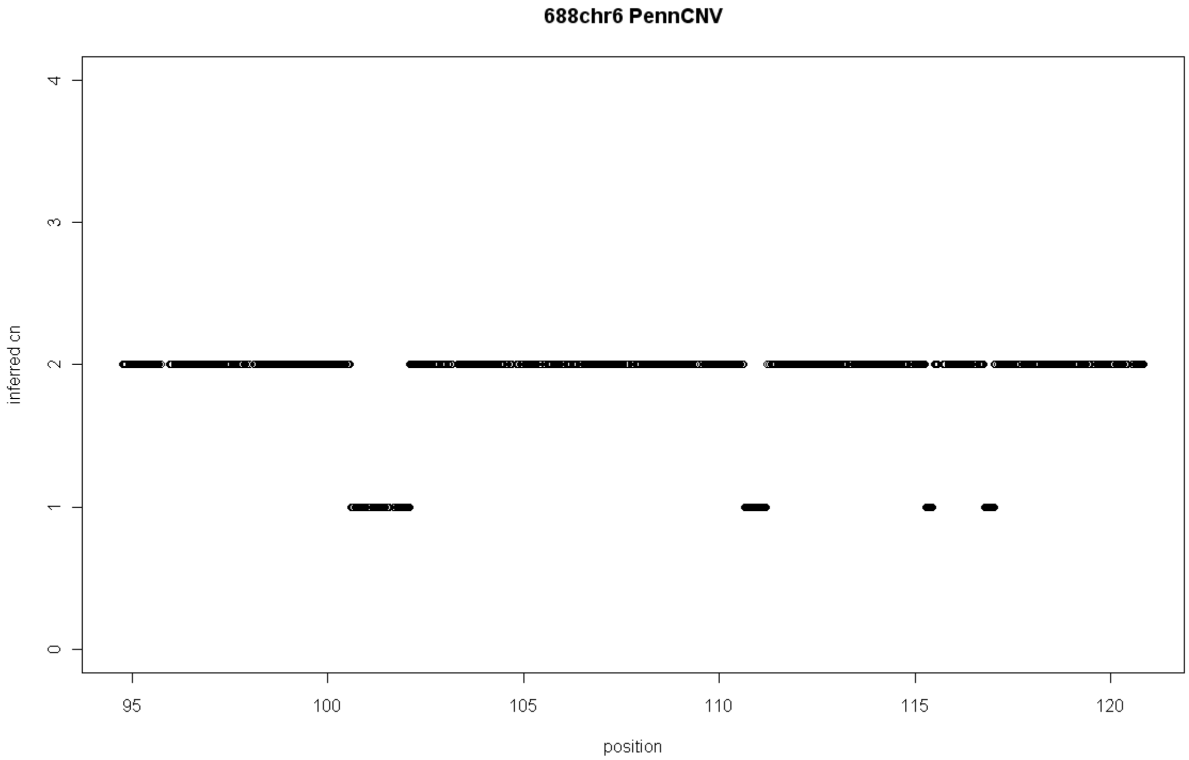

Figure 7: Case 688 chromosome 6: PennCNV

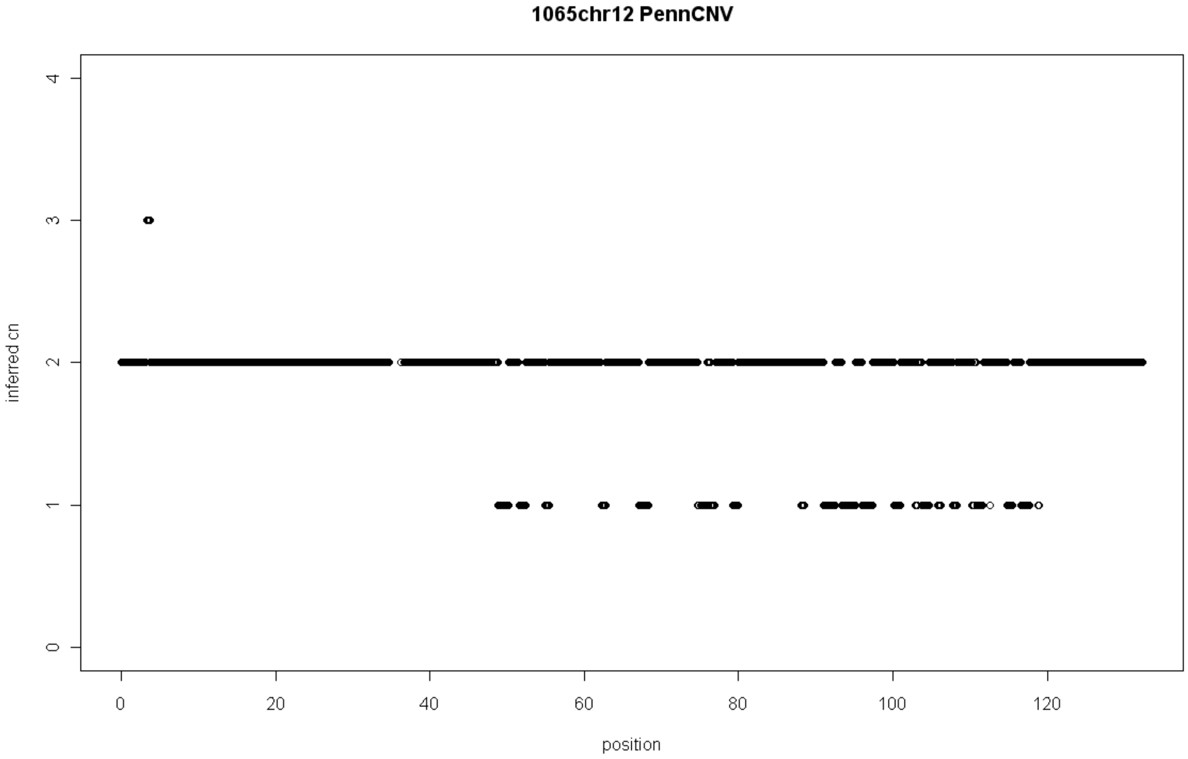

Figure 8: Case 1065 chromosome 12: PennCNV

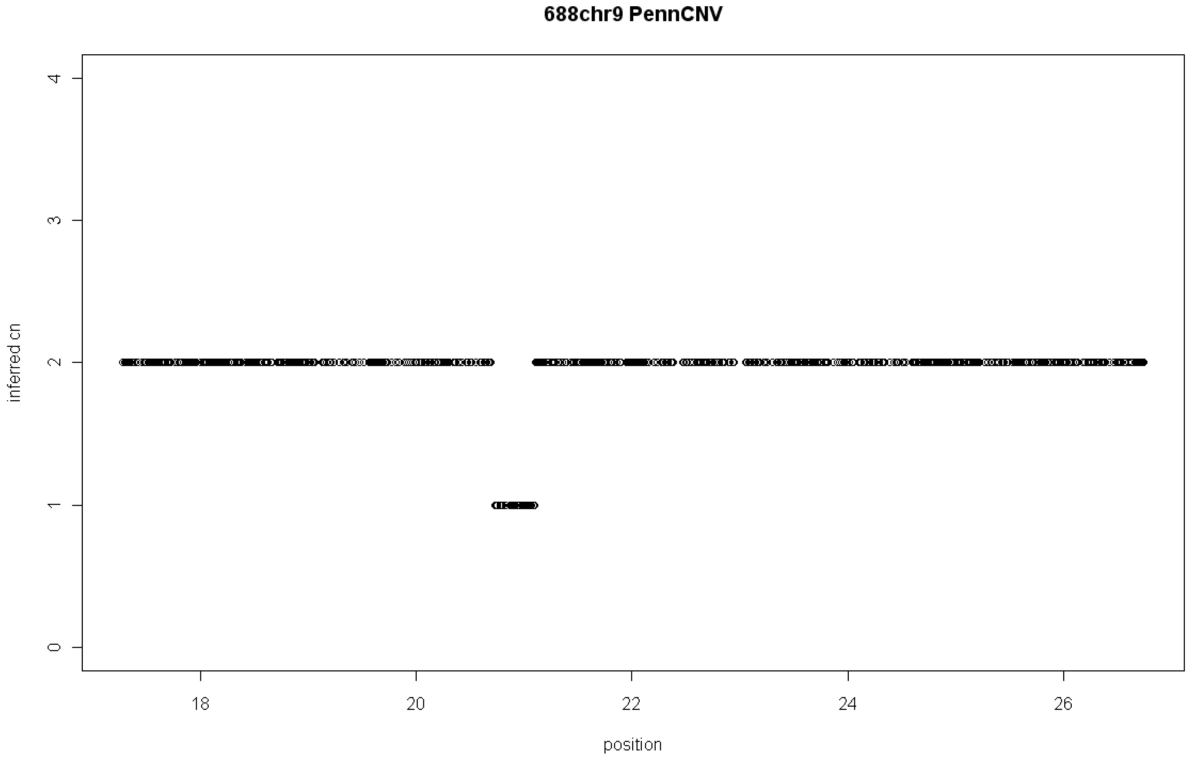

Figure 9: Case 688 chromosome 9: PennCNV
